# Rathke’s Cleft Cyst Leads to Stunted Growth in Children: A Case Report Written With the Help of ChatGPT

**DOI:** 10.7759/cureus.53384

**Published:** 2024-02-01

**Authors:** Huanxiang Huang, Jun Li, Ziqi Li, Yong Xiao, Shousen Wang

**Affiliations:** 1 Department of Neurosurgery, Oriental Hospital Affiliated to Xiamen University, Fuzhou, CHN; 2 Department of Neurosurgery, Fuzhou 900th Hospital, Fuzong Clinical Medical College of Fujian Medical University, Fuzhou, CHN; 3 Department of Neurological Surgery, Central Institute for Mental Health, University of Heidelberg, Heidelberg, DEU

**Keywords:** chat gpt, pituitary function, growth and development, children, rathke’s cleft cyst

## Abstract

In recent times, ChatGPT has become a globally renowned AI tool, revolutionizing academic research by offering innovative methods and opportunities. The integration of AI into various domains is a prevailing topic, focusing on optimizing its utility. This article presents a case study of a child with Rathke's cyst, primarily exhibiting symptoms of growth and developmental delay. The patient's self-perception of stunted growth, coupled with previous assessments indicating partial growth hormone deficiency, prompted further investigation. Laboratory assessments revealed low growth hormone and insulin-like growth factor levels, while imaging disclosed a pituitary lesion. Rathke's cyst was postulated as the probable cause of the growth hormone deficiency. Rathke's cyst remains a rare medical condition with substantial research knowledge gaps. In this article, we synergize ChatGPT responses with a comprehensive case report of a child with Rathke's cyst as the primary symptom-growth and developmental delay. We explore the methods and feasibility of employing ChatGPT within this case report.

## Introduction

Rathke's cleft cysts (RCC) are a rare cystic lesion in the region of the pituitary gland, named after the German anatomist Martin Heinrich Rathke. Typically benign, it occurs in the anterior lobe of the pituitary gland, the front part of the gland. RCC is usually a remnant of the craniopharyngeal duct, a structure in the embryonic development of the pituitary gland. These cysts can be discovered between the ages of 10 and 30, although they may also manifest at other ages. Most RCCs are asymptomatic, especially when small, and may not exhibit obvious clinical signs. However, larger cysts can compress surrounding structures, leading to headaches, reduced vision, visual field deficits, or other neurological symptoms. Additionally, RCC in children can impact their growth and development. Growth hormone (GH) is one of the crucial hormones driving physical development in children. If RCC affects pituitary function, it can lead to reduced growth hormone secretion, resulting in poor growth and development in children, often presenting as slow height growth or short stature. Typically, the diagnosis of RCC involves neuroimaging studies such as magnetic resonance imaging (MRI), and the treatment choice depends on the size and symptoms of the cyst. Smaller cysts may only require regular monitoring, while larger or symptomatic cysts may necessitate surgical removal.

## Case presentation

The patient is a 10-year-old male child who self-reported as shorter in stature compared to his peers. A pituitary lesion was discovered during an examination 10 days ago. He has no history of headaches, dizziness, nausea, vomiting, breast development, visual disturbances, abnormal urination, or limb motor difficulties. A previous assessment at an external clinic suggested a partial growth hormone deficiency. Seeking further diagnosis and treatment, he was admitted to our outpatient department with a provisional diagnosis of "pituitary cyst." Upon admission, his physical examination revealed a height of 129 cm and a weight of 29 kg. According to the "Chinese Children's Growth and Development Standards" (2009 edition) published by the Chinese Ministry of Health, the average height for a 10-year-old child is approximately 143.1 cm, with a standard deviation of about 6.3 cm. The patient's height of 129 cm is approximately 2.24 standard deviations below the average height, indicating that his height is lower than that of most boys his age, although not extremely low.

**Figure 1:**
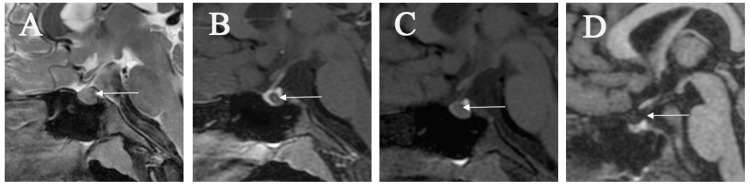
(A) MRI-T2 sagittal view: preoperative MRI-T2 weighted sagittal view shows a well-shaped pituitary gland with nodular hypointense areas within the pituitary (white arrow). (B) MRI-T1 enhanced sagittal view: preoperative pituitary MRI enhanced examination reveals a nodular non-enhancing area between the anterior and posterior lobes of the pituitary (white arrow), measuring approximately 0.6 × 0.6 × 1.3 cm in size. (C) MRI-T1 sagittal view: preoperative MR-T1 weighted image displays a high-signal area in the posterior lobe of the pituitary (thick white arrow), with a T1 hypointense area anterior to the posterior lobe (white arrow). (D) Postoperative pituitary MRI-T1WI sagittal view: shows the disappearance of the previous cystic area within the pituitary, significant reduction in pituitary size, and good imaging of the high-signal area in the posterior lobe of the pituitary (white arrow).

In accordance with the "Evaluation of Height Development in Chinese Children and Adolescents Aged 7 to 18" released by the National Health Commission of the People's Republic of China in 2018, the median height for 10-year-old children is 140.76 cm. The patient's height is approximately 1.68 standard deviations below the normal range, suggesting a moderately lower height level. It's important to note that the "Chinese Children's Growth and Development Standards" (2009 edition) only includes data for children under the age of seven. Laboratory tests revealed the following results: growth hormone (G) and insulin-like growth factor (serum) levels were 0.113 ng/L and 182 (CLIA), respectively. Progesterone (serum) and luteinizing hormone results were 0.32 (CLIA) and 7.00 g/dl (CLIA), respectively. No significant abnormalities were found in other blood tests. Thyroid axis results were as follows: T3 (CLIA) 1.21 ng/ml, T4 (CLIA) 66.5 ng/ml, FT3 (CLIA) 6.54 pmol/ml, and FT4 (CLIA) 16.24 pmol/ml. A pituitary MRI scan, both plain and enhanced, showed that the pituitary stalk was centrally located without apparent signs of compression. The pituitary signal was inhomogeneous, and a cystic abnormal signal was visible inside, with T2WI showing an iso-hypointense signal and T1WI showing an isointense signal. The cyst's dimensions were approximately 0.6 × 0.6 × 1.3 cm (Figures 1A-1C). No other significant signal abnormalities were observed.

Considering the possibility of growth hormone deficiency due to a pituitary cyst, treatment options may include recombinant growth hormone therapy, observation of the pituitary cyst, or recombinant growth hormone therapy after endoscopic transsphenoidal pituitary cyst resection. The patient and their family are strongly willing to undergo surgical treatment. The surgical indications are clear, and relevant preoperative examinations did not reveal any significant contraindications, making the patient suitable for surgical intervention.

During the surgical procedure, a complete bottom of the sella floor was observed. Initially, a puncture was performed to withdraw a colorless, viscous fluid resembling egg white. Subsequently, the dura mater was incised, and the pituitary tissue was dissected longitudinally, leading to the outflow of egg white-like fluid, followed by yellowish gelatinous material. Scraping was performed along the wall of the cyst to minimize the possibility of recurrence. Following admission, the patient's growth hormone (GH) and insulin-like growth factor-1 (IGF-1) levels were monitored for a period of time (the reference range for IGF-1 in children aged 10 to 12 is 64ng/ml-388 ng/ml). An attempt was made to generate a table of patient monitoring data using ChatGPT (Table 1), with December 2, 2020, being the first day of post-surgery. It can be observed that GH exhibited a transient increase after surgery, followed by a gradual decline, with subsequent treatment leading to a gradual recovery to higher levels. Simultaneously, IGF-1 showed a transient decrease post-surgery, followed by a gradual upward trend.

**Table 1:** Postoperative monitoring values of growth hormone (GH) and insulin-like growth factor-1 (IGF-1) in the patient.

Date	GH (ng/ml)	IGF-1 (ng/ml)
2020-11-25	0.113	182
2020-12-02	2.56	172
2020-12-07	0.307	177
2021-06-23	1.44	194
2023-06-20	33.8	-

In June 2021 (eight months post-surgery), the patient underwent a growth hormone stimulation test using 25% arginine at Fuzhou Children's Hospital. Blood samples were collected every 30 minutes, and a total of six measurements were taken. The peak growth hormone (GH) value was 4.19 ng/mL, and the nadir GH value was 0.35 ng/mL. The dynamic GH values were as follows: 4.05 ng/mL (30 minutes), 3.22 ng/mL (60 minutes), 0.60 ng/mL (90 minutes), 0.35 ng/mL (120 minutes), 4.19 ng/mL (150 minutes), and 1.20 ng/mL (180 minutes). A table summarizing these dynamic GH values was created using ChatGPT (Table 2)​​​​​​. 

**Table 2:** Dynamic GH value of the patient growth hormone stimulation experiment. GH: growth hormone.

Time point	GH (ng/ml)
30 minutes	4.05
60 minutes	3.22
90 minutes	0.60
120 minutes	0.35
150 minutes	4.19
180 minutes	1.20

Subsequently, starting from October 30, 2021, the patient initiated long-acting growth hormone treatment with subcutaneous injections before bedtime, at a dosage of 0.17 IU/kg/d once a week. On January 19, 2022, and April 2, 2023, the patient's IGF-1 values were measured at 384.00 ng/ml and 358.00 ng/ml, respectively, showing a significant improvement compared to the pre-treatment levels. During the patient's follow-up for growth assessment, it was noted that in July 2022, the patient's height had reached 140 cm (−1.376 SD), and the bone age examination closely matched the expected changes for the patient's current age. A follow-up MRI was performed three years after the surgery (Figure 1D) and revealed a regression at the original pituitary cyst location with no signs of recurrence. Currently, the patient is in good physical condition, has excellent intellectual abilities, and remains unaffected by RCC and the surgery.

## Discussion

Rathke's cleft cyst (RCC) is a relatively rare condition with a lower incidence compared to other types of brain tumors. Specific incidence data may vary due to regional differences, study populations, and research methods. According to existing studies and literature, the incidence of RCC is estimated to be approximately one to three cases per million people per year. This means that in a specific population in a given region, there may be one to three new cases of RCC discovered each year*. *The data provided by ChatGPT has not been confirmed, and the incidence of symptomatic RCC is indeed relatively rare. Therefore, the exact incidence of this condition remains uncertain. In a previous report by Teramoto et al., subclinical RCC was found in 11.3% of 1000 routine autopsies [1]. For pediatric RCC, the occurrence is even lower, with a prevalence of 3.04% (14/460) in a retrospective analysis of 460 pediatric MRIs conducted by Schmidt [2].

The symptoms of RCC can vary depending on the size of the cyst, its location, and whether it compresses surrounding structures. Some patients may not exhibit any symptoms in the early stages, while others may develop symptoms as the cyst enlarges or affects pituitary function. Common symptoms of RCC include headaches, vision problems, hormonal imbalances, facial and body swelling, pressure symptoms, nausea, and vomiting. The main differential diagnoses for RCC include craniopharyngioma and pituitary adenoma. Summary of distinguishing features among them (Table 3).

**Table 3:** RCC distinguishing table summarized by ChatGPT. RCC: Rathke's cleft cyst.

Disease	Distinguishing features
Rathke's cleft cyst	Originates from Rathke's pouch remnants; often asymptomatic but may cause hormonal imbalances, headaches, or visual disturbances. Typically non-enhancing on MRI.
Craniopharyngioma	Arises from pituitary stalk or nearby tissues; presents with headaches, visual changes, endocrine abnormalities; may have calcifications and can be cystic or solid.
Pituitary adenoma	Originates in pituitary gland; common hormonal disturbances; vision problems due to nearby compression; enhancing lesion on MRI.

In this case, the patient's primary symptoms were related to poor growth and development. The main reasons for this may include: (1) Pituitary compression: the growth of RCC may exert pressure on the pituitary gland, affecting its structure and function. This can disrupt the synthesis and secretion of pituitary hormones, including the production of growth hormones. Growth hormone is a vital hormone that regulates bone and tissue growth. When its secretion is limited, it can lead to impaired growth and development. (2) Increased ventricular pressure: as RCC grows, it may exert pressure on the ventricles, subsequently affecting the flow and absorption of cerebrospinal fluid. Elevated ventricular pressure can lead to cerebrospinal fluid imbalances, resulting in adverse effects on brain structure and function, including impacts on growth and development. This particular factor is not applicable in this case and is incorrect. (3) Pituitary dysfunction: the presence of RCC could lead to overall dysfunction of the pituitary gland, thereby affecting the production and balance of various hormones. Pituitary dysfunction can result in abnormalities in other hormones, such as thyroid hormones and sex hormones, which can in turn impact the growth and development of bones and body tissues.

Furthermore, research suggested that the clinical pituitary dysfunction in the patient might be attributed to granulomatous pituitary inflammation caused by the leakage of cyst contents [3]. In many cases, chronic inflammation is involved in the development of pituitary dysfunction [4]​​​​​​​. For small, asymptomatic RCCs, doctors typically opt for regular MRI monitoring to observe the growth of the cyst and whether it is causing symptoms. If the cyst doesn't significantly increase in size and symptoms are absent or minimal, active treatment is usually unnecessary, and close monitoring suffices. Medication therapy is an optional approach, particularly suitable for slowly growing RCCs. In cases where the cyst has significantly enlarged and is causing symptoms, surgical intervention may be necessary. Surgery can be performed through the transsphenoidal or endoscopic transnasal approach to minimize damage to surrounding structures. For patients who are not suitable candidates for surgery, radiation therapy is another viable option to control cyst growth and alleviate symptoms. For pediatric RCC patients presenting with symptoms of short stature, hormone therapy has shown promising results. Recent research indicates that recombinant growth hormone (rGH) treatment improves height SD scores and does not lead to cyst growth during treatment. Similarly, GnRH analog therapy is effective. ​​Given the potential for spontaneous regression, irreversible pituitary damage, and the risk of recurrence, hormone therapy is a reasonable option for relatively small RCCs [5]. Transsphenoidal surgery remains the preferred choice for symptomatic RCC patients, those with subclinical visual impairment, or those with pituitary dysfunction. It offers excellent outcomes in terms of symptom improvement, preservation of pituitary function, and minimizing complications [6]​​​​​​​.

For the questions we posed in this case report (Table 4)​​​​​​​, ChatGPT provides reasonably good output, especially in providing informative and well-written general paragraphs. It can offer useful information and comprehensive descriptions of relevant topics. Furthermore, it can design tables based on the provided data. However, when addressing specific questions, ChatGPT tends to offer guidance and general principles rather than addressing the question directly. This suggests that it may lack in-depth question understanding in certain cases and may not provide detailed or specific answers. Regarding data-related questions, ChatGPT often fails to output real or verifiable data. This is because its responses are based on information encountered in its training data, rather than on real-time or specific database data. Therefore, in questions involving specific numbers or real data, ChatGPT's responses may lack accuracy and credibility. Additionally, ChatGPT appears to have some latency. In situations where it has not been trained on a sufficient amount of new text data, it may not fully comprehend the latest information or events. 

**Table 4:** Questions were provided to ChatGPT.

Introduction of RCC
Does RCC in children affect their growth and development?
How many standard deviations does a Chinese 10-year-old boy with a height of 129 cm fall below the standard height?
The prevalence of RCC.
Symptoms of RCC.
What diseases do RCC need to be differentiated and diagnosed with.
The causes of growth and development disorders caused by RCC.
The treatment plan for RCC with growth disorders as the main manifestation and a size of approximately 0.6 × 0.6 × 1.3 cm.

**Figure 2:**
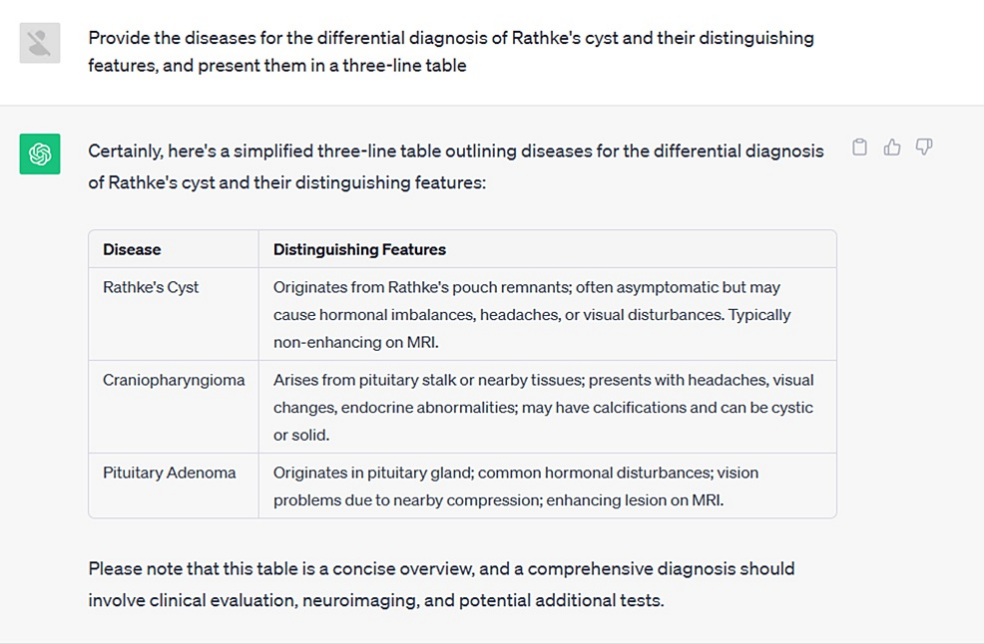
Image of a three-row table generated by ChatGPT.

Thus, when responding to time-sensitive questions, ChatGPT's responses may not be timely or accurate enough. It's worth noting that when we requested ChatGPT to provide a three-column table, it appeared to provide a three-row table (Figure 2). This might be related to the limitations of its language model training.​​​​​​​ To overcome some of ChatGPT's limitations, we need to view it as a smart assistant rather than an ultimate expert. Especially in academic and professional applications, we should consider its outputs as preliminary insights to stimulate thought, rather than definitive conclusions. When faced with critically important questions, the best practice is to seek the assistance and guidance of domain experts who can further research and validate the information. Furthermore, during ChatGPT's training, ensuring it has access to a diverse and rich "diet" of data is crucial. Providing it with a broader, more up-to-date, and accurate dataset is like feeding it intellectually nutritious food, which helps enhance its understanding of questions and provide more accurate responses.

## Conclusions

This case report discusses a 10-year-old male child with Rathke's cleft cyst (RCC) that led to stunted growth and explores the potential applications and feasibility of using ChatGPT in academic research. RCC may compress and damage the pituitary gland, thereby interfering with the production and secretion of growth hormones. Treatment options depend on the cyst's size and symptoms, with surgical removal and hormone therapy being common choices. However, ChatGPT has limitations in answering questions, particularly in providing specific data and addressing individual cases. Therefore, the reasonable utilization of ChatGPT in academic applications offers new avenues for medical research and clinical decision-making. The choice of treatment plans and monitoring methods is crucial for patients.
